# *InCliniGene* enables high-throughput and comprehensive *in vivo* clonal tracking toward clinical genomics data integration

**DOI:** 10.1093/database/baad069

**Published:** 2023-11-02

**Authors:** Ivan Merelli, Stefano Beretta, Daniela Cesana, Alessandro Gennari, Fabrizio Benedicenti, Giulio Spinozzi, Daniele Cesini, Eugenio Montini, Daniele D’Agostino, Andrea Calabria

**Affiliations:** San Raffaele Telethon Institute for Gene Therapy, IRCCS Ospedale San Raffaele, Via Olgettina 60, Milano 20132, Italy; San Raffaele Telethon Institute for Gene Therapy, IRCCS Ospedale San Raffaele, Via Olgettina 60, Milano 20132, Italy; San Raffaele Telethon Institute for Gene Therapy, IRCCS Ospedale San Raffaele, Via Olgettina 60, Milano 20132, Italy; San Raffaele Telethon Institute for Gene Therapy, IRCCS Ospedale San Raffaele, Via Olgettina 60, Milano 20132, Italy; San Raffaele Telethon Institute for Gene Therapy, IRCCS Ospedale San Raffaele, Via Olgettina 60, Milano 20132, Italy; Centro Nazionale Analisi Fotogrammi (CNAF), Istituto Nazionale di Fisica Nucleare, Viale Carlo Berti Pichat 6/2, Bologna 40127, Italy; San Raffaele Telethon Institute for Gene Therapy, IRCCS Ospedale San Raffaele, Via Olgettina 60, Milano 20132, Italy; Dipartimento di Informatica, Bioingegneria, Robotica e Ingegneria dei Sistemi (DIBRIS), Università degli Studi di Genova, Viale Causa 13, Genoa 16145, Italy; Institute of Biomedical Technologies, Italian National Research Council, Via Fratelli Cervi 93, Segrate (MI) 20054, Italy; San Raffaele Telethon Institute for Gene Therapy, IRCCS Ospedale San Raffaele, Via Olgettina 60, Milano 20132, Italy; San Raffaele Telethon Institute for Gene Therapy, IRCCS Ospedale San Raffaele, Via Olgettina 60, Milano 20132, Italy

## Abstract

High-throughput clonal tracking in patients under hematopoietic stem cell gene therapy with integrating vector is instrumental in assessing bio-safety and efficacy. Monitoring the fate of millions of transplanted clones and their progeny across differentiation and proliferation over time leverages the identification of the vector integration sites, used as surrogates of clonal identity. Although γ-tracking retroviral insertion sites (γ-TRIS) is the state-of-the-art algorithm for clonal identification, the computational drawbacks in the tracking algorithm, based on a combinatorial all-versus-all strategy, limit its use in clinical studies with several thousands of samples per patient. We developed the first clonal tracking graph database, *InCliniGene* (https://github.com/calabrialab/InCliniGene), that imports the output files of γ-TRIS and generates the graph of clones (nodes) connected by arches if two nodes share common genomic features as defined by the γ-TRIS rules. Embedding both clonal data and their connections in the graph, *InCliniGene* can track all clones longitudinally over samples through data queries that fully explore the graph. This approach resulted in being highly accurate and scalable. We validated *InCliniGene* using an *in vitro* dataset, specifically designed to mimic clinical cases, and tested the accuracy and precision. *InCliniGene* allows extensive use of γ-TRIS in large gene therapy clinical applications and naturally realizes the full data integration of molecular and genomics data, clinical and treatment measurements and genomic annotations. Further extensions of *InCliniGene* with data federation and with application programming interface will support data mining toward precision, personalized and predictive medicine in gene therapy.

**Database URL:**  https://github.com/calabrialab/InCliniGene

## Introduction

One of the most intriguing and challenging research directions in hematopoietic stem cell (HSC) biology is monitoring *in vivo* the fate of millions of clones with the lens of genomics (1). Although several molecular biology technologies are approaching this aim, most of them are feasible only in animal models (2–5) due to ethical issues or uncontrolled off-target mutations such as gene editing with cellular barcoding, whereas only few of them are viable in humans, such as gene therapy with viral vector gene addition strategy (6–8). In gene therapy, clinical applications based on engineered viral vectors (both γ-retroviral vectors or lentiviral vectors) (6, 9), HSCs are harvested from diseased patients and, upon genetic correction through integrating a functional copy of the defective gene using viral vector, are transplanted into patients to fully reconstitute the hematopoietic system and ensure life-long efficacy. Hence, the integration of the viral vector, which occurs semi-randomly along the genome of the target cells, is a stable genetic mark of each independent engrafted HSC, inherited by all its cell progeny and maintained in the process of differentiation and proliferation. For this reason, vector integration sites (ISs) can be used as identifiers of the cellular identity of each transplanted and engrafted HSC clone to track its fate over time and tissues. Moreover, ISs allow for studying the safety and long-term efficacy of the treatment, excluding insertional mutagenesis (10, 11). Therefore, ISs have been recently exploited for dissecting the hematopoietic reconstitution *in vivo* as evolutionary processes involving millions of clones and cells in distinct tissues at cellular and molecular levels, enabling for the first time a deeper knowledge of the hematopoietic system after transplantation and long term in human (12–14). Unfortunately, the clonal tracking characterization in clinical trials is an expensive study generating huge amounts of genomic data to be processed, analyzed and interpreted, with consequently computational drawbacks to handle.

**Figure 1.**
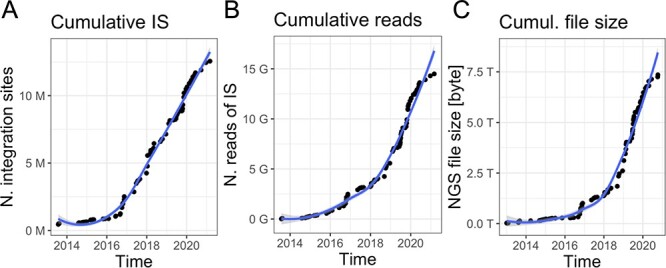
Data volume of clonal tracking studies in clinical trials for gene therapy applications at SR-Tiget by (A) integration sites, (B) sequencing reads of IS and (C) NGS file size (sequencing pools from several technologies). ‘Number of reads’ refers to the overall number of sequencing reads; ‘Number of IS’ refers to the number of integration sites identified in the samples, corresponding to clones. ‘File size’ corresponds to the size of the files of the NGS pools (gzip compression).

Gene therapy patients are monitored over decade-long life-span post-autologous transplantation (13, 15–17). Peripheral blood and bone marrow samples are periodically collected at every predefined months (from 1 to 12) and samples are subjected to PCR-based procedures (14, 18, 19) to retrieve vector ISs and sequenced with high-throughput platforms such as Illumina paired-ends technology. Indeed, the volume of data is constantly increasing together with the number of samples per patient and time (an example in Figure 1 is related to our ongoing clinical studies), generating scalability issues. Although in the early days of sequencing (roughly 10 years ago), the output file size was relatively large but <10 GB per file, nowadays each sequencing library is mainly processed by the highest throughput platforms, like the Nova-seq, thus adding >250 GB of data for each update. These observations led us expecting a rapid data volume accumulation as we are experiencing in our clinical studies in which we reached > 7TB of raw data (Figure 1C) in the last years. Downstream raw sequencing reads processing, the scalability issue will be integrating and analyzing all data for longitudinal clonal tracking.

The most advanced tool for IS identification is γ-tracking retroviral insertion sites (γ-TRIS) (20), assessed by the best precision and recall compared to other tools. γ-TRIS can include clones landing in repetitive elements or low-complexity regions beyond the IS mapping in single genomic loci which are returned by most of the other tools (21–27).

**Figure 2.**
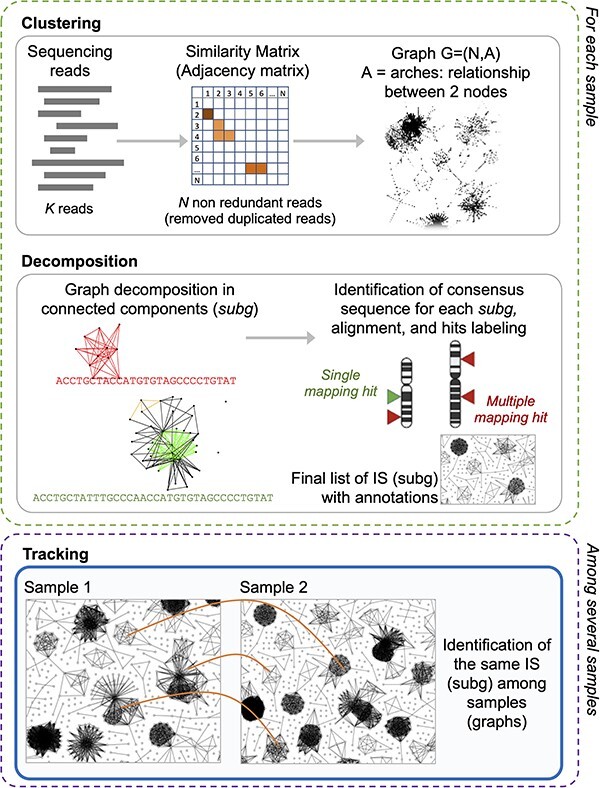
γ-TRIS main steps: (1) clustering, (2) decomposition, and (3) tracking. Steps 1 and 2 are performed for each sample in the sequencing library, whereas step 3 only generates the tracking matrix (indeed it may be run at the library level or even as collections of several libraries, as expected in clinical trial monitoring).

Briefly summarizing the main steps (Figure 2), the algorithm of γ-TRIS is designed to process IS sequencing reads derived from each single sample, and starts by *clustering* reads by their sequence similarity without aligning them to a reference genome, and generates the graph of the retrieved clusters (homogeneous sequences representing a putative IS) in which each node is a unique read and edges connect nodes by their observed similarity. As a result, the graph presents highly connected sub-graphs (putative ISs) that are then analyzed (*decomposition* step) to obtain the core sub-graphs and thus the final list of ISs (called *subg*). For simplicity, hereafter we will refer to synonymous ISs and clones as well as subg and sub-graphs. Once extracted the sub-graphs from all the samples, γ-TRIS moves to the next step of the analysis aimed at identifying the same clone (here represented by a *subg*) across all the distinct samples. This step is called *tracking* and the current approach is based on the comparison of each *subg* of a sample versus all the other subg of the other samples. At the end of the step, γ-TRIS returns a data matrix of ISs (rows) observed in each analyzed sample (columns) and quantified with the corresponding number of reads (cell values).

The tracking step is the most computationally intensive phase of the whole analysis since (i) it is based on a combinatorial comparison of each subg of a sample against all the other subg in the other samples, (ii) all the data are stored, so far, in files, thus increasing I/O operations and overhead lead to huge execution times and (iii) it needs to be run at every insertion of new data (i.e. new sequencing libraries/pools and new patient’s follow-ups).

Indeed, in the tracking step of γ-TRIS, both the number of samples that can be analyzed and the number of IS retrieved [for example, highly polyclonal samples processed with new efficient and sensitive methods of IS retrieval (14)] deeply limit the scalability of the tool and, therefore, the broader use of this method in whole clinical trials.

To overcome this limitation, we propose a novel approach based on the use of graph databases (hereafter graph DB) in order to avoid the direct pairwise comparison of ISs to be analyzed.

In fact, ISs, genomic positions and labels can be stored as nodes and linked together depending on the results of the decomposition step. With this modeling approach, we can consider only IS pairs that share at least a genomic position (*pos* node) or one label (*label* node). A graph DB is able to provide this information straightforwardly compared to a relational database approach. This is due to the fact that a graph DB makes links between nodes as first class citizens (28) instead of using join operations as realized in relational databases. The consequence is that tracking an IS across connected samples requires only the navigation of existing links, and indeed, from a theoretical point of view, this approach could scale-up very rapidly.

In the following sections, we describe how we designed and developed a new graph DB solution for clonal tracking and validated our approach on *in vitro* data, here generated to replicate real-case ISs. In particular, our validation dataset is composed of a mix of known ISs with random unknown ISs at several proportions designed to mimic a wide range of datasets from a high diversity (that is, many clones with an equal distribution abundance) to a low diversity (that is, few clones dominating in terms of abundance). If correctly structured, the queries to our graph DB would return the same known IS tracked across all the distinct samples, independently of the abundance of the IS and the number of samples. We called our graph DB *InCliniGene* (integrated clinical genomics database), and we released it on GitHub together with all the necessary documentation and tools for importing new data (https://github.com/calabrialab/InCliniGene).

In the field of computational biology, only a few examples of graph DB have been already proposed, for example, to efficiently integrate heterogeneous repositories (29), or for specific application domains such as chemical informatics with Bio4j (30), biomolecular pathways analysis with *Reactome* (31) or chromatin conformation capture experiments (32).

The Neo4j (https://www.neo4j.com) graph database management system has been adopted in nearly all these works because it represents today the *de facto* standard for this data model. For this reason, we exploited Neo4j in our solution.

### Data description

The dataset structure has been directly derived from the application domain in which DNA samples are collected from gene therapy patients at different time points. When patients are enrolled in a gene therapy clinical trial, they are visited by clinicians and a set of clinical variables (such as patient age, type of mutations, ethnicity, etc.) are collected by assigning a unique identifier to each patient and anonymized in all subsequent procedures. Then, each patient undergoes bone marrow harvest that is treated with *ex vivo* gene therapy and, during this process a small amount of DNA material (usually left-over after transplantation) is used for clonal identification. After the time of transplantation, patients are monitored in predefined months by blood and bone marrow harvest, followed by IS analysis. In detail, the blood sample is processed to isolate specific cell lineages, and the cellular DNA is extracted for IS retrieval, accounting ∼ 12–18 samples per harvest. Then, each piece of information of the biological sample is linked to the source patient, with the corresponding treatment details, and the experimental details (e.g. amount of DNA, PCR method used to capture viral/host cellular junctions, etc.). Finally, samples undergo PCR amplification and sequencing by the multiplexed library with Illumina platform, to produce a large file of reads in FASTQ format (extended version of the FASTA format to embed Quality data per base, acquiring the name by the original FASTP program) that is analyzed with γ-TRIS to identify ISs.

In the first step of γ-TRIS, for each sample, sequences are connected in a graph structure according to their similarity, that is, nodes are the input reads and edges represent the edit distance among the corresponding sequences, and then the graph is decomposed to produce highly connected clusters (called subgraphs or *subg*) representing ISs. For each subgraph, a consensus sequence is computed, and all the genomic annotations after alignments are added. Moreover, genomic labels are processed to annotate the IS as unique or landing in repetitive elements. In the latter case, the subgraph presents several hits on the target genome and no unique genomic regions can be used as representative. The decomposition step will then consolidate the IS identity by analyzing all connected components of each *subg* to eventually decompose the cluster into distinct ones, thus obtaining independent ISs. The final step of γ- TRIS, *tracking*, compares all the *subg*s by processing their features (genomic and repeat labels) to identify the same *subg* observed in different samples and thus to track the same IS across observations.

The *tracking* step of γ-TRIS is based on specific rules, previously designed to correctly assign two *subg*, belonging to distinct samples, to the same IS. Here we summarize the rules implemented in γ-TRIS to assign two *subgs* of distinct samples to the same IS: given two samples, *A* and *B*, and one IS for each sample, *IS_A_* and *IS_B_*, the two ISs will be assigned to the same clonal identity if and only if:

- IS*_A_* and IS*_B_* correspond to the same single genomic position;
- IS*_A_* and IS*_B_* share at least 50% of the genomic positions;
- IS*_A_* and IS*_B_* share at least one label and one genomic position;
- IS*_A_* and IS*_B_* share the same genomic position having *max* alignment score.

Since our database is designed to handle the tracking phase of γ-TRIS, and potentially of all other IS software, we needed to port the same rules into our import procedure, enabling the generation of all the links in the graph connecting independent *subg*s identified from different samples. These rules need to be translated during the data import phase into the graph DB.

### The clonal tracking graph structure

**Figure 3.**
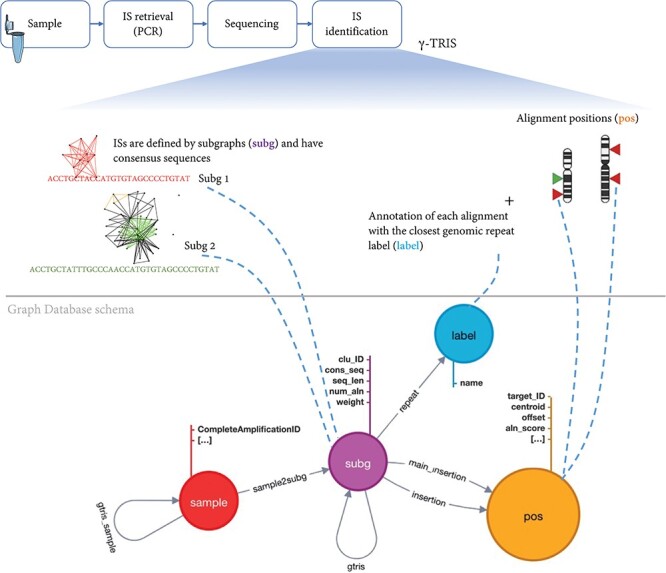
Schema of the graph database archiving clonal data obtained from ISs using γ-TRIS. A node *subg* represents a subgraph and collects information about a source IS and the consensus sequence. One *sample* node is connected to many *subg* nodes, while a *subg* node is linked to one and only one sample. Subgraph annotations/labels are represented by *label* nodes, as well as the genomic loci of the ISs on the reference genome are represented by *pos* nodes. The relationship connecting a *subg* and a label is called *repeat*, while the link between a subg and a pos is called *insertion*. This information is all included in the files representing the input of the tracking step. On the other hand, γ-TRIS links are computed during the tracking step using the rules described in ‘Data description’ section and incrementally, i.e. new *gtris* links are computed and added only when a new sample is inserted in the database. These links represent the novel approach for speeding up and scaling the tracking step analysis.

To archive the results of a gene therapy application, we first need to design a database schema that collects all the main elements of the experimental, clinical procedure and molecular observations (in this context, the vector IS) after the analysis of the requirements. Our main observation (entity) is a sample that also inherits the information about the source Project, PatientID, Tissue, Timepoint and all the other experimental details connected to a single sample. A sample is identified by a key. Each IS is observed in one sample and potentially can be observed (recaptured) in different samples, thus leading to a shared IS across samples. Each IS is identified by the genomic coordinates, orientation and by alternative alignments. Moreover, each IS retails the quantification of the source number of sequencing reads or DNA fragments. Once γ-TRIS returned the list of IS as *subg*, a series of technical information is added as fields including the consensus sequence, the alignment scores for each hit, the number of alignments, the value of each distinct clonal quantification approach (for example, sequence count as *weight*), the consensus sequence, the list of alignments (*insertion*) with their scores and genomic positions (*pos*) among which a *main insertion* is selected as a representative if not recognized as a *repeat* (labeled and classified by the repeat source family). In the tracking phase of γ-TRIS, additional features are added, such as the connections between two *samples* (with the name *gtrissample*) if they share at least one *subg*; if two *subg* are connected in the tracking phase then the *gtris* link is created among *subg*. Given the description of these entities and their connections, we have been able to design our database schema for the graph DB structured. Given the analysis of the requirements, we designed the graph DB as a directed graph composed of four types of nodes (namely, *sample, subg, label* and *pos*) and having relationships connecting nodes that share the same feature (Figure 3).

Each sample, along with patient and experimental information, is represented by a node of type *sample*. These nodes are involved in two types of relationships: *sample2subg* and *gtris_sample*. The first one connects each *sample* to all the identified ISs (*subg* nodes) during the first step of γ-TRIS, while the *gtris_sample* relationship connects different *sample* according to the tracking step of the γ-TRIS workflow. Subgraphs are represented by *subg* nodes, which collect information about the source IS and the consensus sequence. Subgraph annotations/labels are identified by *label* nodes. The relationship connecting *subg* and *label* is called *repeat*. Each *subg* is connected to *pos* that represent the genomic loci of the ISs on the reference genome. Two distinct types of relationships connect *subg* and *pos* nodes: *insertion* and *main_insertion*. If the consensus sequence of a subgraph aligns to several genomic regions (thus describing a repeat) that cannot be disambiguated through the selection of the representative element, the node *subg* connects many *pos* nodes through the relationship *insertion*. On the other hand, if a subgraph aligns a single genomic region or has a single dominant alignment (corresponding to the best alignment in terms of length and score, with values that are at least twice the second hit), the relationships among the corresponding *subg* and *pos* nodes are called *main_insertion*.

All the information except *gtris* and *gtris_sample* and are already present in the file produced by γ-TRIS for each sample and are processed by a Java application to be inserted in the graph DB, the starting point to speed-up the tracking step (see ‘Methods’ section ‘Data Import into *InCliniGene*’ for details).

On the contrary, the relationships *gtris* and *gtris_sample* are computed during the insertion of a sample with respect to the samples already present in the DB. A further description of the process implemented in the abovementioned Java application for creating these two types of links is present in the ‘Methods’ section.

### Large-scale validation assay

Here we present an experimental dataset as a real-case scenario in which γ-TRIS struggled to perform the tracking step and to finalize the data matrix for downstream clonal tracking analyses. The dataset's extensive size, including the number of columns (samples), data volume, and the count of clones (independent ISs or *subg*) significantly affected γ-TRIS’s computational capabilities. This impact necessitated all-versus-all comparisons and ultimately rendered the process unfeasible. Addressing this challenge with our innovative solution will demonstrate the practical application of the graph DB.

Gene therapy clonal tracking data may present two opposite scenarios: (i) an extreme polyclonal case, in which many clones (>500) are present in the sample(s) and each one has a relatively small number of cells (<10–20); (ii) an extreme oligoclonal scenario, in which very few clones are present (<50) and some of the clones has the vast majority of cells, thus fully dominating the population. In the first case, the population diversity, often measured with the Shannon/Renyi diversity index (33) will be maximum, while in the latter case will be minimum (close to zero).

To mimic the real-case range of clonal tracking data, from a highly polyclonal condition to a highly oligoclonal one, we designed an experimental dataset in which we mixed two cell lines with known ISs in a population of random ISs (Table 1). The proportion of the mixes (that we can hereon call *samples*) has been designed to replicate the polyclonal condition, thus the abundance of the ISs derived from the cell lines was similar to the random ISs. Then, we increased the abundance of the known IS up to completely dominating the sample, and thus these samples will contain only a few ISs beyond the known ones whose abundance is much higher than the random ISs.

**Table 1.** Experimental design of the large-scale validation assay (14). The three cell lines are reported with their names (CEM1, CEM3 and JY) and the number of known or random IS. The standard conditions (mixes of the cell lines) are reported in the next columns, in which the cell line with a known IS (CEM1) is diluted in the random JY cell line. We classify a dilution as oligoclonal, marked with a star * symbol close to the mix ID, if the proportion of JY cells is <50%, otherwise polyclonal. The number of expected ISs varies from 7, in the dilutions with 0% of JY cells, to several hundreds of ISs, where JY cells are close to 50%, or >5 ISs00 if JY cells are >50%

		Standards (dilutions)								
Cell line	N. IS	L*	M*	N*	O*	P	Q	R	S	T
**CEM1**	**1 known IS**	70%	60%	50%	25%	12.50%	7.50%	0.75%	0.08%	0%
**CEM6**	**6 known IS**	30%	30%	30%	30%	30%	30%	30%	30%	30%
**JY (Random)**	**N. random IS**	0%	10%	20%	45%	58%	63%	69%	70%	70%

The experimental set-up of the mixed cell lines and dilutions was then tested on 10 independent PCR methodologies to identify the best approach to retrieve ISs and quantify their clonal abundance. Moreover, each sample was performed in three biological replicates and three technical replicates. Indeed, all the different samples compose a particularly large dataset in which known ISs are well-tracked at different abundances across dilutions. Each sample was sequenced by Illumina platform and processed with γ-TRIS.

Given the need of analyzing this dataset and producing the clonal tracking matrix to be processed for downstream analyses [e.g. by using ISAnalytics (34)], we decided to use this case study as a limiting scenario to be addressed by our graph DB solution. The number of samples included in the experimental data was 856, sequenced in 2 lanes with HiSeq Illumina paired-end technology, obtaining a total number of 324 495 807 reads, corresponding to >130 GB of data. The average number of final reads belonging to ISs was 67 469 (excluding low-quality samples, with number of reads < 10). The number of final ISs identified by the graph DB was 1 151 671, having an overall number of 71 879 749 reads supporting them.

Unfortunately, due to the size of the data, in terms of the number of samples and clones, the computation of the tracking matrix required combining all ISs from all the samples (second step of clonal tracking), resulting in unfeasible for γ-TRIS.

### The graph DB

The instance of the resulting Neo4j graph DB for our validation assay dataset is composed of 13 200 373 nodes and 22 276 481 links between them. The size of the dump in graphML format is 6.1 GB.

On the DB graph nodes, the number of samples with valid read alignments and successfully imported is 765, resulting in 1 153 970 *subg*. The remaining missing samples are related to failed PCRs due to experimental problems in which no DNA fragments were present to obtain valid reads, thus returning empty sequencing files. The number of distinct *pos* is 12 044 596, with 1042 labels. In detail, each sample contains from 11 up to 7767 *subg*, while the number of samples with >1000 *subg* is 346. Every *subg* is associated with at least 1 *pos*, but, in the most redundant case, it shows 205 975 possible matches. In general, 91% of the *subg* is associated with a single genomic position, but 4380 have more than 100 correspondences.

The number of *gtris* links computed during the data import is 4 513 646. Among them, 89% are links between *subg* and a single *pos*, resulting unique genomic loci directly associated with a single IS. The remaining 501 096 links connect *subg* with more complex patterns, obtained by translating the γ-TRIS rules for the database import procedure. More in detail, we designed the four main cases as described in the ‘Data description’ section, and our matched results on the dataset are the following: Case (i): 4 012 548 nodes (89%); Case (ii): 211 712 nodes; Case (iii): 270 120 nodes; Case (iv): 19 264 nodes. Moreover, these links are unequally distributed: only 311 651 *subg* are connected to each other and, among them, 57% *subg* have a single connection with another *subg*, while 7501 have more than 100 connections. The maximum value is 896 connections.

The numbers of *pos* nodes and *gtris* links are reciprocally related but not linearly. For example, the *subg* having 896 connections has only 15 genomic positions, while the *subg* having 205 975 possible matches has no *gtris* links.

### Clonal expansion dataset

Here we present another experimental dataset designed to replicate clonal expansions, called *VA20*. In this case, we mixed four cell lines, each of them with known ISs, with the JY cell line with a random and unknown number of IS. The mixes were generated at different proportions (Table 2) to replicate several hypothetical situations of expanding clones with different numbers of ISs per clone (named Vector Copy Number). We here introduce this experiment since γ-TRIS correctly computed both the steps and returned the output clonal matrix. Indeed, we will use this dataset to validate the output of *InCliniGene* by performing a direct comparison.

**Table 2.** Experimental design of the clonal expansion (VA20) assay. The five cell lines are reported with their IDs (A–D, and JY) and the number of known or random IS. The standard conditions (mixes of the cell lines with the mix ID as column header) are reported in columns. Each cell line is diluted in proportion to the corresponding percentage reported in its column. The oligoclonal mixes are marked with a star * symbol close to the mix ID, using the same classification criteria previously reported: if the proportion of the JY cells is <50% is considered oligoclonal, otherwise polyclonal

		Standards (dilutions/mixes) [%]																
Cell line	N. IS	1*	2	3	4	5	6	7	8*	9*	10*	11*	12*	13*	14*	15*	16*	17
A	1	25	5	1	0.5	0.1	0.05	5	5	5	100	0	0	0	40	*3*	8	25
B	3	25	5	1	0.5	0.1	0.05	25	50	75	0	100	0	0	3	40	25	8
C	6	25	5	1	0.5	0.1	0.05	5	5	5	0	0	100	0	8	25	40	3
D	10	25	5	1	0.5	0.1	0.05	5	5	5	0	0	0	100	40	25	3	8
JY	*N*	0	80	96	98	99.6	99.8	60	35	10	0	0	0	0	9	7	24	56

For our *in vitro* dataset *VA20*, we obtained 54 DNA samples which were then sequenced by NovaSeq Illumina paired-ends chemical reagents, producing a total of 522 791 964 reads corresponding to a file size (gzip compressed) of > 106 GB, then processed by γ-TRIS to identify the list of ISs, each one with its abundance. The sequencing raw reads files were filtered by quality and then were subjected to the removal of non-informative data (such as sequences belonging to the internal control band or not presenting vector sequences) for a total of 117 241 081 reads. The distribution of the raw reads per sample showed an average of 2 171 131 reads with a standard deviation of 862 538 sequences. The final number of reads belonging to the identified ISs was on average 882 057 (std. dev. 366 997). The size of this dataset and its data volume, in particular in the number of samples, allowed γ-TRIS to perform all the three steps, generating both the list of ISs (by the clustering and deconvolution steps) and the tracking matrix (via the tracking step). The overall number of ISs, summed along the distinct 54 samples, was 86 173 (from 20 to 6063), with a variable IS abundance from 1 to 1 658 159 reads based on the specific dilution. The final data matrix resulted in sparse data with a modest file size (<1 MB) after running all steps of γ-TRIS. Given the small size of the output, we used this case scenario for precision assessment.

The clonal expansion dataset (*VA20*) imported into *InCliniGene* resulted in the following number of nodes and arches: node *sample* were 120, *pos* 829 589, *label* 860, *subg* 86 061; edges among nodes were 86 061 for relation *sample2subg*, 1331 for *gtris_sample*, 871 113 for *insertion*, 44 231 for *repeat*, 51 795 for *gtris* and 1763 for *main_insertion*. In terms of data comparison with the results obtained by γ-TRIS, we used one sequencing lane, thus analyzing 54 samples instead of the whole database of 120 nodes.

## Results

We analyzed the graph DB solution under distinct perspectives: (i) to validate the method in terms of tracking accuracy using the small validation assay (called *VA20*) for which γ-TRIS returned the output matrix, thus allowing us to perform a direct comparison between γ-TRIS output and *InCliniGene* output; (ii) to measure the feasibility and to assess the scalability of *InCliniGene* with the large-scale validation assay towards clinical applications; (iii) to test the performance in terms of data retrieval using an equivalent and alternative solution based on relational database.

### Validating the graph DB approach

To validate if our solution was in agreement with the output of γ-TRIS, we used a small *in vitro* dataset, *VA20*, for which γ-TRIS could complete the overall analysis and return the list of ISs. The choice of using an *in vitro* dataset for this aim is based on the purpose of (i) having a real-case ground truth, not just a simulated dataset, and (ii) exploiting a dataset generated to cover a wide range of real-case scenarios contained in small and controlled scale (likewise, the large validation dataset previously described).

We compared γ-TRIS and *InCliniGene* by analyzing the final list of ISs tracked among all the samples of the dataset, separating ISs landing in repeats and ISs with unique mapping. This choice followed two main reasons: (i) ISs in repeats are marked with a random ID by each software, indeed linking the random numbers between γ-TRIS and *InCliniGene* is meaningless; (ii) nevertheless, we needed to quantitatively compare both the locus identity (operation valid only in ISs with unique genomic positions), and the IS abundance (precisely for IS in unique genomic loci whether with summary statistics for ISs in repeats). The comparison exploited the same sparse matrix file format, typically returned by IS identification tools having clones as rows and samples as columns, see ‘Methods’ section for the query to build the matrix from *InCliniGene*. For the ISs labeled with a genomic locus, we compared both the number of those returned by the tools, their ID and their quantification. For the ISs labeled as repetitive elements, we compared the overall amount of ISs and their cumulative abundance.

Our results (see the GitHub repository for the code generating the comparative analysis, folder *compare_dataset*) showed that the number of ISs was almost identical between the two softwares with an average percentage difference < 0.2%, and showed a perfect match on the overall number of sequencing reads (IS abundance) < 0.35% of the unique reads was different in terms of selected representative genomic position while the remaining > 99.65% of clones was exact at the identical genomic base.

These results validate the solution of using the graph DB as a method for archiving and querying clonal data, and the module for generating the final clonal tracking matrix, the aim of the project. Having assessed the precision of the graph DB method, we can move to the larger dataset presented above to test the scalability and the challenge of the computational performances.

### Testing clonal tracking in *in vitro* large-volume datasets

We measured the running time to import all data of samples from the large-scale *in vitro* dataset in the graph DB, exploiting the import script (see ‘Methods’ section for the query and the computational infrastructure details). This operation was accomplished in ∼ 80 h. The most time-consuming step was related to the creation of *gtris* links, because *gtris_sample* ones are a consequence of connecting one or more *subg* belonging to two samples. The creation of nodes and links of the other types (e.g. *pos* and *insertion*) instead was driven by the parsing of the file containing the data for each sample.

The first sample was inserted in 10 s, the last one in 12 min (Table 3). The first sample creates 28 *subg* related to 129 *pos*, the last one 3052 *subg* related to 12 112 *pos*. The first sample did not have any *gtris* link, the last one 760. These numbers seem to suggest that the processing time is proportional to all three elements, but the number of pos is particularly relevant. The creation of all the elements different from the *gtris* links (as stated above we can disregard the *gtris_sample* ones) has a nearly constant time. This is because the creation of a new, single node or a new, single link requires a nearly constant amount of time in the graph DB. On the contrary, the analysis for possibly creating the *gtris* links requires considering all the *subg* that share at least one *pos* with the *subg* we are inserting. Therefore, the complexity of the query is proportional to the number of retrieved results, which can vary from a few up to several thousand. This is because more pos nodes are linked to the subg of a sample, and more subg nodes already present in the graph DB need to be extracted and analyzed for calculating gtris links. This is shown by the characteristics of the most time-consuming sample, which required >2 h to be inserted. It was composed of 3025 *subg* and 361 314 *pos*. This last number was mainly due to a single *subg*, which was linked to 205 975 *pos*. However, the number of *gtris* links involving the *subg* of this sample was only 6978. Another sample, processed in 11 min, was composed of 2305 *subg* and 27 156 *pos*, but it was involved in a comparable number of *gtris* links (i.e. 6532).

**Table 3.** Characteristics of some samples and import times in the graph DB. The import time is mainly proportional to the number of *subg* nodes together with the sum of insertion links created by a sample. This is due to the fact that, for each *subg*, there is the need to query the graph DB in order to extract *subg* candidate to be linked with it with a gtris link. The execution time of these queries is proportional to the number of insertion links

Sampl/e	Time	*subg* nodes	New links *insertion*	New links *gtris*
1	10 s	28	129	0
2	12 s	31	121	2686
3	26 s	68	105	8528
4	11 min	2305	27 156	6532
5	12 min	3052	12 112	760
6	122 min	3025	361 314	6978

Table 3 summarizes the above data. We can conclude that the insertion time is mainly proportional to the number and the complexity of the queries necessary to create possible new gtris links. The number is proportional to the number of *subg* contained in a sample, while the complexity is proportional to the number of insertion links. In general, if we exclude exceptional situations, the time to process a file containing a sample ranges from a dozen seconds up to a few minutes.

It is further clear that inserting a new sample in the database requires more time with respect to the first samples already inserted, because of the presence of more *subg* sharing with the available *pos*. However, we would like to stress the fact that it mainly depends on the number of the *pos* involved. For example, let us consider two comparable samples, one inserted as second, one as 564*th*. The first one composed of 31 *subg*, 121 *pos* and 2686 *gtris* links, while the second one having 68 *subg*, 105 *pos*, and 8528 *gtris* links. The first one requires 12 s to be inserted, while the second one 26 s, considering that the number of resulting *gtris* is much bigger than the sample requiring 2 h.

We also measured the export time using our query script (see ‘Querying the graph DB to extract IS matrix’ section) to obtain the final data matrix for tracking clones, and we obtained a total time of 12 h. This time remained constant even after having added new samples. We are currently developing optimization strategies to speed up this evaluation by storing some partial results in the database and calculating the rows in parallel.

Under the biological lens, the experimental design of the large-scale validation assay shows in each dilution a cell line (CEM6) at a constant proportion (30%), meaning that the six known ISs of the CEM6 will be equally represented at the same abundance for each mix and across all samples. Indeed, we can check the consistency of the results in terms of (i) intra-sample abundance for the 6 ISs, and (ii) tracking efficiency and inter-sample abundance. Moreover, since only with *InCliniGene* will be possible to analyze the results of this experiment including ISs landing in repeats, we will further validate and compare the results of IS abundance with the output of another common tool, VISPA2 (23), demonstrating the advantage of using γ-TRIS and the importance of analyzing the data with *InCliniGene*.

We compared our results with one of the most recent IS retrieval tools, VISPA2 (23), in terms of clonal quantification for the IS landing in a low-complexity region. The clonal abundance in VISPA2 was realized through the estimate of the number of cells per clone by SonicLength (35), since it represents the most accurate quantification method; the quantification is called fragment estimate. In γ-TRIS and *InCliniGene*, we used the number of distinct shearing sites (that is, the number of different genomic fragments per clone) and the attached unique molecular identifier (UMI). In both cases, we then applied a relative percentage of each IS to the total amount of observed/estimated cells in each sample. Querying *InCliniGene* for the CEM6 ISs, we obtained all six distinct known ISs that were tracked among the distinct experiments and across the different experimental conditions. Five out of six resulted mapped in unique genomic loci, thus not requiring the label repeat, whereas only one IS resulted mapped into a repetitive element.

The summary tables with all the IS abundance quantifications are available in the folder *validation_assay_large* of the repository. The expected abundance of each CEM6 IS is 9.4%, independent of the experiment or replicate. We considered here the most accurate experiment (called *LMv2-I*), to run our comparative analyses. Our results showed that the CEM6 IS at the genomic locus chr16:28 497 498 was quantified by VISPA2 with an average of 2.4% (std. dev. 0.64) across the reliable dilutions (meaning above the detection limit of the experimental procedure). On the other hand, γ-TRIS combined with *InCliniGene* quantified the same IS at an average abundance of 8.4% (std. dev. 0.89). These results confirmed that γ-TRIS improved the precision in IS quantification by including all read landing in low-complexity regions which instead are discarded by VISPA2. These findings led us to confirm the hypothesis that *InCliniGene* overcome the initial computational challenges of γ-TRIS maintaining the same precision.

Furthermore, we released in the repository (within the folder *utils)* an additional plugin to convert any clonal tracking data, in the form of a sparse matrix, in the JSON file format and import the data into *InCliniGene*. This feature enables a broader usability and flexibility of our graph DB, not only restricted to γ-TRIS but rather extended to all potential IS tools and their output.

### Graph DB or relational DB?

In order to provide a further quantitative demonstration of the efficacy of the graph DB paradigm in clonal tracking applications, we compared our solution with the use of the traditional relational database. Here we selected a MySQL database system (release details in ‘Methods’ section) since other tools for IS retrieval already adopted this solution (19).

**Figure 4.**
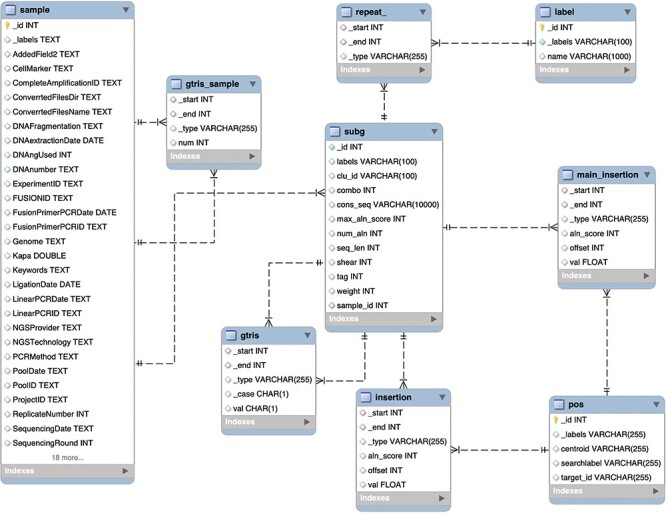
Schema of the relational database MySQL archiving clonal data obtained from integration sites using γ-TRIS. Similarly to the Graph DB structure (3), the entity-relationship diagram of the requirements described in the ‘Data description’ section has been converted in this table schema.

We designed and tested specific *ad hoc* queries for distinct operators on both the graph DB and the relational DB, and we benchmarked the performances, inspired by the work of Cheng (36). This is because our goal was to assess the performance in data retrieval using the explicitly stored links between nodes with respect to the need to perform multiple joins. We disregarded the aspects related to data import, and in particular we created the relational DB assuming that the *gtris* and *gtris_sample* relationships were already calculated. The resulting database structure is described in the entity relationship (ER) diagram of Figure 4.

**Table 4.** Queries on clonal data both for relational DB and graph DB

Queries	Operations	SQL language	CYPHER language
**01**	projection join filter	select U.clu_id from ((subg U join sample S on U.sample_id = S._id) join insertion I on U._id = I._start) join pos P on I._end = P._id where P.searchlabel = ‘chr2_24546570ʹ and S.DNAnumber = ‘R’	match (s:sample)—(u:subg)-[:insertion]-(p:pos) where p.searchlabel = ‘chr2_24546570ʹ and s.DNAnumber = ‘R’ return u.clu_id
**02**	projection join aggregation	select S.DNAnumber, count(U._id) from subg U join sample S on U.sample_id = S._id group by S.DNAnumber	match (s:sample)—(u:subg) return s.DNAnumber, count(u)
**03**	projection join aggregation	select S.DNAnumber, count(P._id) from ((subg U join sample S on U.sample_id = S._id) join insertion I on U._id = I._start) join pos P on I._end = P._id group by S.DNAnumber	match (s:sample)—(u:subg)-[:insertion]-(p:pos) return s.DNAnumber, count(p)
**04**	projection join aggregation order by	select P.target_id, count(distinct U.clu_id) from (pos P join insertion I on P._id = I._end) join subg U on I._start = U._id group by P.target_id order by P.target_id	match (u:subg)-[:insertion]-(p:pos) return p.target_id, count(distinct u.clu_id) order by p.target_id
**05**	projection join filter aggregation	select count(B.clu_id) from subg B where B.clu_id in (select U.clu_id from (pos P join insertion I on P._id = I._end) join subg U on I._start = U._id group by U.clu_id having count(P._id) > 2)	match (u:subg)-[i:insertion]-(p:pos) with u.clu_id as uci, count(p) as num_i where num_i > 2 return count(uci)
**06**	projection join filter aggregation	select S.DNAnumber, count(B.clu_id) from subg B join sample S on B.sample_id = S._id where B.clu_id in (select U.clu_id from (pos P join insertion I on P._id = I._end) join subg U on I._start = U._id group by U.clu_id having count(P._id) > 2) group by S.DNAnumber order by S.DNAnumber	match (s:sample)—(u:subg)-[i:insertion]-(p:pos) with u.clu_id as uci, count(p) as num_i, s.DNAnumber as sample_type where num_i > 2 return sample_type, count(uci) order by sample_type

The queries used to test the performances on both datasets are representative of the most common primitive operations, including *projection, aggregation, join, filter*, and *order by. Projection* queries are used for selecting which attribute(s) the queries should return. *Join* queries combine data columns from one or more tables in the relational database, whereas in the graph database the join operations are not needed because relationships are embedded within the data as an entity. *Aggregation* queries are designed for grouping together the values of multiple rows, and *Order by* queries allow custom sorting of the results (36). In Table 4, we reported both the SQL query and the Cypher translation, referring to the MySQL and Neo4j solution, respectively.

The performances of MySQL and Neo4j databases were measured using the computational infrastructure described in the ‘Computational environment’ section under the following metrics:

• Execution time: the query processing time of the same queries, in SQL or Cypher language;

• Memory utilization: the difference in terms of memory occupied, between the query execution state and the resting state.

The value of these metrics is computed as the average of five repeated (independent) measurements in order to have the most accurate results. The details of the computational environment and software version are reported in ‘Computational environment’ section.

**Figure 5.**
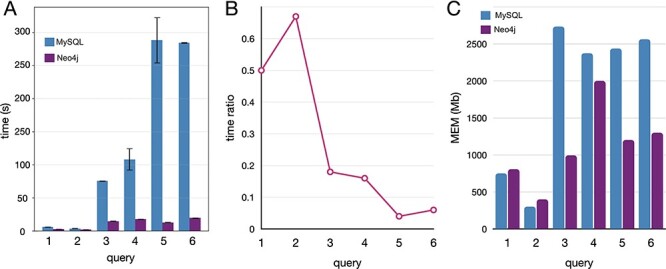
Comparative benchmarks of the computational performances between Neo4J and MySQL databases applied to real-case queries, here reported with five instances covering different operations. (A) Time in seconds, with variance (whiskers) for the replicates, scaled to *log*_10_; (B) time ratio between Neo4j and MySQL (query time Neo4j/MySQL); (C) the memory utilization in megabytes.

Executed the queries in both databases, Neo4j resulted much faster and with lower memory requirements (Figure 5). In both DBMS, the performances depend on the amount of data traversed by the query. Nevertheless, MySQL query time and memory usage increase faster than Neo4j. For this reason, query 2, which requires a join of only two tables in MySQL, performed better than the other queries. The same behavior is observed in Neo4j, although the join operations are not executed. Queries 1 and 3 traverse the same volume of data, but query 1 has a filter operation while query 3 an aggregation operation. Our results from the performances obtained by executing queries 1 and 3 suggest that the filter operations are less demanding than the aggregation, both in terms of time and memory. Queries 4, 5 and 6 traverse the whole database (more than 13 million entities) and perform a combination of complex operations like filter, aggregation and order by. The time ratio (Figure 5B) is much lower (indicating a major improvement in Neo4j) than in queries 1–3 since the amount of data is larger, leveraging on the performances with a negative impact in the MySQL instance.

## Discussion

In current gene therapy applications, we are now facing critical data management and processing issues due to the accumulation of terabytes of genomics data, as represented in 1. Computational challenges have indeed risen and must be addressed with state-of-the-art technologies, such as social networking and new database structures. In this work, we presented *InCliniGene*, a new solution to overcome the computational limitations in tracking clones according to the results achieved with γ-TRIS, the most comprehensive tool for IS identification in gene therapy datasets based on integrating viral vectors. Moreover, *InCliniGene* represents the first database of whole sample ISs in *in vivo* applications, collecting all IS rather than a selection of curated clones (37–40).

With InCliniGene, we were able to scale up the usage of γ-TRIS to large-volume data such as the validation assay here illustrated that is composed of > 850 samples. Indeed, our solution is finally making practical performing clonal tracking studies in whole clinical trials and potentially comparing the dynamics of the hematopoietic reconstitution *in vivo* after transplantation, characterizing the stem cell activity in terms of differentiation and proliferation (HSC biology) also using ISs landing in repeats.

The key aspect of *InCliniGene* is the adoption of a graph DB data model. Why to use it for clonal tracking? The whole world of big data is moving towards dynamics solutions to both archive data and connect the corresponding structures to minimize all the downstream efforts in data mining or simply querying. In this context, graph DB deeply supported social networking systems, requiring high scalability, to both store and query data. Similarly to social networks, γ-TRIS composes genomic sequences in a graph that is further processed to identify ISs. In fact, the corresponding clones will have their own evolution and dynamics *in vivo* in patients under gene therapy and followed-up for many years. This scenario can be approached to a population in which the transplanted stem cell clones are the entities (species) that naturally compete for their survival and progression through differentiation and proliferation during the hematopoietic reconstitution. The parallelism between the two application domains, combined with the native structure of the clonal data in γ-TRIS, oriented our choice to use the same tools of social networking systems, starting from the graph DB. Only with a graph-based approach, all nodes (IS or clones) are connected to each other via links that are computed once and explicitly stored. Querying this data structure resulted in fast and highly scalable operations. These characteristics hold true also for a dynamic scenario as the presented one, i.e. where new data are constantly imported in the graph DB and the whole graph is updated to connect the new data instances. In fact, in our application domain of gene therapy, we need to follow up with patients even for more than a decade, processing at predefined time intervals a set of samples, potentially including all newly enrolled patients in the study/protocol. Moreover, the graph DB will process more insert/import operations than other transactions. Once the import is completed and once all new connections are drawn, all ISs are automatically connected, and tracking clones across samples (time, tissues, lineages, etc.) is directly returned by querying the data (as we described in the query ‘Querying the graph DB to extract IS matrix’).

Whether Neo4j is the best graph database management system rather if better solutions exist is out the scope of this work and will be addressed in further studies.

Another important aspect that we figured out is related to network analysis: what types of biological questions can we effectively address with network-based metrics? Is it possible to derive any biological readouts for the assessment of safety and efficacy using clonal tracking data and their connectivity? Any new proprieties of HSC evolution or activity?

We used our validation dataset to identify potential directions. Results from these analyses (data not shown) were not satisfactory and we identified the potential explanation for the type of data. In particular, here we used *in vitro* data design to answer specific questions on the most precise PCR method for IS retrieval and quantification, with replicated samples and in a serial dilution setting. Beyond these questions, no further conclusions could be expected in terms of safety assessment or HSC biology. Indeed, centrality eigenvector or community structure metrics could at most confirm the expected highest connectivity of the known ISs across all samples, providing weak suggestions on how to setup and configure the centrality indexes in time-series clonal tracking gene therapy applications to identify any potentially expanding worrisome clones, or on how to study lineage restriction during the hematopoietic reconstitution.

Nevertheless, we can figure out some interesting directions while using our solution in a whole clinical trial, in particular using genes and their structural or functional annotations. The reason why using genes is directly connected to the application domain. In gene therapy, safety issues are mainly related to insertional mutagenesis, meaning that the viral vector, originally engineered to release the therapeutic transgene in the genome of the diseased cells, integrates and deregulates the closest gene favoring genotoxic events (41) that could lead to oncogenesis. The clones harboring the ISs in deregulated genes are positively selected over time and are tracked with abnormal growth. Indeed, gene network analysis would support readouts of genotoxicity by generating a gene network of the targeted genes, connected as they are close by topological proximity by nuclear architecture, thus the popularity of a gene would be directly related to the conformation of the nucleus. The main reason for this choice is that some viral vectors, such as lentiviral vectors, integrate close to the nuclear membrane (42–44) and current tools for studying genomic hotspots of integration are still missing the topological data in humans, although some studies in murine models have already demonstrated how to combine ISs with chromosomal architecture for genotoxicity (45, 46). Only by using a graph database it would be possible to integrate not only genomic annotations and features (such as genes and functions) but also additional high-throughput genomic data such as Hi-C conformation capture sequencing. Likely, graph database tools already exist embedding and analyzing Hi-C data (47–49), enabling easier integration with our *InCliniGene* solution.

An alternative use of the gene network is exploiting the already known structure of gene regulatory networks (50) and using each patient gene dataset over time to identify the progression of the patient genes (that is, in case of a clonal selection, only a few ISs harbored by the expanding clones will be recaptured) and thus studying the clonal selection process. Clonal selection can be seen as network perturbation and the analysis of the (gene) network centrality would eventually suggest if critical genes are selected and worrisome trajectories towards oncogenesis.

Beyond the gene networks, can we use the eigenvector centrality to study the importance of single clones instead of functional genes? Translating this question in the field of HSC biology, can we identify any relevant stem cell clone (or pool of clones) that is mainly sustaining the hematopoietic reconstitution or the homeostasis after transplantation? This question is moving our perspective even further of using the graph database and its properties, and these answers will be concretely useful not only for the assessment of the safety but also to unravel paramount goals of *in vivo* human stem cell research, finally improving the protocols for gene therapy treatment. Some studies already approached transcriptomics analysis of single-cell datasets using network centrality for cellular heterogeneity (51), regulation of hematopoiesis (52) or plasticity (53). Indeed, we envisage using *InCliniGene* and the graph metrics (eigenvector centrality, community structure detection, etc.) for unraveling hematopoietic lineage dynamics.

### Conclusions and perspectives

In gene therapy, no publicly available human databases exist for clonal tracking although IS analysis is instrumental for the registration and commercialization of the treatment as requested by regulatory authorities. Only some instances of manually curated databases of ISs have been released (37–40) with major limitations such as (i) containing very few ISs and not the whole repertoire observed in patients, (ii) the ISs are not tracked over time, thus their information cannot be used for data progression, and (iii) no treatment information is reported rather only some genomic annotations.

*InCliniGene* instead overcomes these limitations and a future public exposition with a web interface and API would allow extensive use not only for clonal tracking studies within the same institute but also favoring data integration of multiple institutes and organizations, thus creating the first resource collecting molecular data of several cohorts of patients and diseases. Neo4j is already predisposed to be data federated with the *fabric* component.

Moreover, the combination of genomic data of clones with other high-throughput sequencing data (such as Hi-C data or expression data, as previously discussed), and with clinical and treatment data, will fully realize the vertical data integration. The final aim of vertical data integration will be clinical genomics, moving clonal tracking studies toward personalized and predictive medicine. A direct consequence of the clinical genomics perspective is having a unique resource to be used in combination with machine-learning methods to improve precision medicine in targeted therapies, such as gene therapy, and support new findings in HSC biology.

## Methods

### Experimental datasets for *in vitro* validation

The method for vector IS retrieval, called Sonication Linker mediated (SLiM)-PCR, was tested on 9 *ad-hoc* DNA standards. DNA standards were generated by mixing the genomic DNA extracted from two cell line clones, named CEM1 and CEM6, carrying one and six different Lentiviral Vector (LV) IS, respectively in known genomic positions, with the genomic DNA of a cell line, named JY, harboring lentiviral IS randomly distributed in unknown positions of the genome, with average vector copies per cell (diploid genome) of 1.8. CEM6 was maintained at 30% across the DNA standards, while CEM1 was diluted from 70% to 0% and, conversely, JY from 0% to 70% to simulate oligoclonal to polyclonal conditions.

The procedure, applied in nine replicates for each of the DNA standards, consists of the following steps: (i) fragmentation by sonication of the DNA, (ii) ligation of the fragments to a linker cassette (LC), (iii) two consecutive rounds of PCR, to specifically amplify vector/cellular–genome junctions, by using primers annealing to the vector genome end (Long Terminal Repeats, LTR) and the LC. Primers contain DNA barcodes, that allow univocal barcoding of all the SLiM-PCR replicates, and sequencing adapters that allow multiplexed sequencing on Illumina sequencers.

Sequencing data was then analyzed by VISPA2 (23) and γ- TRIS (20) to identify ISs by their genomic coordinates (and labels if landing into genomic positions with repetitive elements) and to quantify their abundance. Since the genomic DNA of the cells sharing the same IS will be sheared, the resulting genomic fragments will likely have different lengths, one per cell. Indeed, counting the number of different fragment lengths will reflect the abundance of the clone, unless two or more cells will have the same fragment length. SLiM-PCR can retrieve and identify single genomic fragments thanks to the LC attached prior amplification. Nevertheless, no experimental procedures can avoid the quantification problem of having the same fragment length for two cells of the same IS. We used the statistical method SonicLength (21) to estimate the size (abundance as number of cells) of each clone. For all the DNA standards we measured the abundance of each of the CEM IS and we calculated the deviation from the expected values, to evaluate the SLiM-PCR performance and sensitivity.

### Computational environment

The performance comparison has been performed on a workstation with the following specifications: CPU Intel Xeon with 2.4 Ghz processor 8 core; 125 GB RAM, 1000 GB hard disk; operating system CentOS Linux 7; MySQL Community Server 8.0.30; Neo4j 4.4.9–1. Tests have been realized with the default configuration settings in both DBMS, with minor parameter editing: *innodb_buffer_pool_size *= 16384 M in MySQL; *dbmsmemory.heap.max_size *= 16384 M in Neo4j.

Benchmark procedure required in MySQL to free memory before each query, repeating the database restart procedure for each query. The MySQL restart procedure has not been included in the evaluation of the computational resources. This approach was required to prevent MySQL from caching useful data for reducing the computation time of the next queries. Query execution time in MySQL was measured using Linux time command, whereas in Neo4j it is automatically printed along with the query results.

### Data import into *InCliniGene*

Here we summarize the import procedure of the γ-TRIS output into *InCliniGene*. γ-TRIS output is a JSON file containing information about the IS identified, along with their alignment positions on the reference genome and their labels. Since γ-TRIS processes the first two steps (clustering and decomposition) by sample, the import program is designed to run for each sample, thus for every update (new samples) all the corresponding results can be imported into the *InCliniGene* database. We developed the procedure in Java application described below and the workflow consists in parsing the output file and building the graph at run-time.

#### Parsing

The class CluParser includes the commands to parse the file; the process consists in reading one IS at time and saving all the information in an Java object of the class Insertion, which collects IS data that will constitute the *subg* attributes, the list (named *targetlist*) of all the genomic positions the insertion aligns and the insertion labels (string masked). *targetlist* is a list of objects of the class Target, these objects collect the identifier of each position along with their information (namely *centroid, offset, alignment score*).

#### Graph building

The generation of the graph is carried out by the AddInsertion method of the Insertion class. At the first instance of the import, related to the first IS, a node of type *sample* is created, describing the sample analyzed by γ-TRIS. This *sample* node has a *UniqueID at-tribute*, which is an identifier retrieved from the γ-TRIS output file name. Further information is retrieved from a separate metadata file and added to *sample* node. Then, the parsed data are added to the graph database, creating one node of type *subg* for the insertion, several *pos* nodes for each genomic position collected in targetlist and *label* nodes for each *label*. The relationships connecting nodes are then created: *sample2subg* between *sample* and *subg* nodes, *repeat* between *subg* and *label* nodes, *insertion* between *subg* and *pos* nodes. Whenever the *subg* node connects several *pos* nodes (thus becoming a repeat) through several *insertion* relationships, a predominant *pos* node is searched, and, if present, it is connected to the *subg* node also with the relationship *main_insertion*. This search process parses the *pos* attribute *aln_score*: in the targetlist, if the highest value of *aln_score* differs for more than 30% from the next score, the *pos* node of the top hit is connected through a *main_insertion* relationship to the *subg* node. The code here reported shows a scratch to open a connection to Neo4j and run the query in Cypher.

command1 = "MATCH(ss:sample {UniqueID: \"+id+"\"})\ n";
command1 = command1.concat("MERGE
(s:subg {clu_id: \"" + cluid + "\", " + "cons_seq: \"" + cons_seq + "\", "+ "num_aln: " + num_aln + ", " +
"cons_seq: \"" + cons_seq + "\", " + "max_aln_score: " + max_aln_score + ", " + "seq_len: " + seq_len + ", " +
"weight: " + weight + "})\ n");
command1 = command1.concat("MERGE (ss)-[:sample2subg]->(s)");
try (Session session = d.session()) { session.run(command1); }

At the end of this step, all the information has been retrieved from the γ-TRIS output file. Relationships among *subg* nodes, (named *gtris*) are subsequently built from scratch, comparing IS (*subg*) from all the samples in the database. This comparison is carried out following the γ-TRIS rules mentioned earlier. When two *subg* nodes are connected through the *gtris* relationship, consequently also the *sample* nodes they derive from are connected by the relationship *gtris_sample*.

### Querying the graph DB to extract IS matrix

We designed and implemented a query to obtain a data matrix of ISs in the data format commonly used to clonal tracking in which samples are in columns, clones are in rows and are annotated with their genomic coordinates or repetitive labels (in the case of IS surround- ing a repetitive element), and cell values are the quantification of the IS observed in that sample. The available clonal quantifications are based on (i) sequencing reads count, (ii) counts of shearing sited (that is, the number of distinct observed fragment lengths from sequencing reads), (iii) the number of UMI observed and (iv) the combination of counts of UMI and shearing sites.

To generate the matrix of IS, we developed a Java application that executes several query loops for each IS in which *InCliniGene* follows the graph and all IS connections. Most part of the matrix construction is done by the table method of the TableCreator class. This method requires three arguments: driver, threshold and wlabel. The driver allows the connection to the Neo4j database; threshold is a float variable to shrink the *main_insertions* definitions (default set to 0.3) and wlabel defines the type of quantification desired among ‘weight’, ‘shear’, ‘tag’, ‘combo’ or ‘all’. In case the wlabel parameter is equal to ‘all’, the four matrices will be implemented, otherwise just the one specified. In detail, in every step of the table method, wlabel defines the data to be retrieved from the database and the quantification type (a specific one or all) to be built. The first query retrieves the *UniqueID* of all the samples in the database, these data are then used to build the header of the four matrixes, which is: *ID, Chr, Pos, Strand, numsubg, numexp, subg*, plus all the samples *UniqueID*.

Then, data regarding the IS (*subg*) is retrieved: if wlabel is equal to ‘all’ are retrieved the following *subg* attributes: *clu_id* (the unique identifier), *weight, shear* (accounting for the number of distinct shearing sites per IS, that is the distinct fragment lengths), *tag* (accounting the unique molecular identifiers) and *combo* (accounting both the UMI and the number of shearing sites); otherwise are retrieved *clu_id* and only the specified attribute. These data are saved into four hash-tables, each one collecting the *clu_id* and the associated specific attributes (stored in an array).

Once collected, all the IS (*subg*) data, the connections among *subg* nodes are explored. Given a *subg* node, in this example called ‘candidate1’ and obtained by extracting one *clu_id* from the array, the query retrieves all the *subg* nodes directly connected to candidate1 through the *gtris* relationship. Each of the *subg*nodes identified in this way is then compared to ‘candidate1’, looking at the *pos* nodes they are connected to, in order to see if both align on the same position or a different one. If the *subg* node has only one connection to *pos* nodes, the link is straightforward, otherwise the *main_insertion* (if present) is considered. This process is repeated propagating the comparison also for the *subg* nodes which connect ‘candidate1’ through a second-degree connection (subg—[gtris]—subg—[gtris]—candidate1).

At the end of the propagation, all the *subg* nodes retrieved are removed from the array, avoiding reconsidering the same node twice, in this way the array size reduces drastically at every propagation. Finally, the hash tables collecting the data are saved as one row of the IS matrix, and the propagation process is repeated for each next *subg*.

### Analysis and comparison of the tools

The comparison of the validation assay dataset was done by a custom R script that allowed (i) to import both the γ-TRIS matrix and the *InCliniGene* matrix; (ii) to adjust the data formatting by converting the chromosome nomenclature, and minor edits; and (iii) to intersect the raw names and column names of both solutions to analyze the overlap by *dplyR*. The code of the R script together with the data is available in the GitHub repository of the project (https://github.com/calabrialab/InCliniGene).

## Availability of source code

Lists the following:

- Project name: *InCliniGene* (Integrated Clinical Genomics).
- Project home page: https://github.com/calabrialab/InCliniGene.
- Operating system(s): Platform independent.
- Programming language: Java, Cypher, R.
- License: GNU GPL. Any restrictions to use by non-academics: license needed.

## Availability of supporting data and materials

The datasets supporting the results of this article are available in the GitHub repository (https://github.com/calabrialab/InCliniGene) and in the NCBI SRA archive (BioProject: PRJNA886489).
